# Supplementary material for: Embracing heterogeneity: coalescing the Tree of Life and the future of phylogenomics
Source: PeerJ. 2019 Feb 14;7:e6399. doi: 10.7717/peerj.6399 (PMC6378093; doi:10.7717/peerj.6399)
Supplement: Supplemental Information 2 [file peerj-07-6399-s002.pdf]

Supplementary data for Bravo et al., "Embracing heterogeneity: building the Tree of Life and the future of phylogenomics" for *PeerJ*.

Compilation of data in Supplementary Table S1. We assembled 164 phylogenomic data sets from 2004 to 2017 to explore their size and scope and change over time. This list of data sets is not comprehensive. We searched four journals for phylogenomic data sets via keyword "phylogenomics" and the journal title using Web of Science v.5.27. On December 1, 2017, we searched *Molecular Phylogenetics and Evolution*, *Systematic Biology*, and *Molecular Biology and Evolution.* On January 12, 2018 we searched *Proceedings of the Royal Society of London Series B*, again using keyword "phylogenomics". We recognize that we may have captured additional papers using keyword "phylogenom*" or similar, but time constraints prevented addition of many more references. In addition, authors of this paper added further citations based on their familiarity with the literature.

We counted either the number of amino acids or the number of nucleotides in the full alignment (Alignment length [bp or aa]) as reported. We captured analyses that included multiple sequences per species by recording the number of samples, which refers to the total number of rows in a data matrix, as well as the total number of species analyzed. We are aware that many papers analyze matrices with missing data; in our draft table (available on request) we recorded the extent of missing data when easily determined from the paper but otherwise analyzed the data sets as if they were complete.

Criteria for inclusion in the data set: We only included papers that used next-generation sequencing (NGS) methods, whether for amplicon sequencing, transcriptomes, hybrid capture, whole-genome sequencing or other approaches. We also included a few early papers using expressed sequence tags (ESTs) when available. We considered only papers using nuclear genes; no mitogenome-only or chloroplast DNA-only papers were included. We only considered data sets dealing with sequence-based loci, sensu Brito & Edwards (2009), namely data sets composed of markers, each of which is a segment of DNA. We did not consider Rad-seq or other data sets that were distilled into collections of SNPs prior to phylogenetic analysis (Leaché et al. 2017). When papers analyzed multiple data sets, we usually included only the 'main' analysis. Papers from which it was difficult to extract the relevant data were not included.

A living Supplementary Table S1 can be found here. We encourage the community to enrich this dataset by adding relevant references that are consistent with the criteria for inclusion stated above.

Full link:
https://docs.google.com/spreadsheets/d/18UKsxen9CRiyeUtcRP_B1II6UrGD_JWA8cpcnykck6A/edit#gid=0

References

Brito, P., Edwards, S. V. 2009. Multilocus phylogeography and phylogenetics using sequence-based markers. Genetica 135: 439–455.

Leaché, A. D., Oaks, J. R. 2017. The Utility of Single Nucleotide Polymorphism (SNP) Data in Phylogenetics. Annual Review of Ecology, Evolution, and Systematics 48: 69–84.
